# Supplementary material for: Comparative Genomics and Pathogenicity Analysis of Two Bacterial Symbionts of Entomopathogenic Nematodes: The Role of the GroEL Protein in Virulence
Source: Microorganisms. 2022 Feb 22;10(3):486. doi: 10.3390/microorganisms10030486 (PMC8950339; doi:10.3390/microorganisms10030486)
Supplement: Supplementary file 1 [file microorganisms-10-00486-s001.zip › Supplementary_Information_Microorganism.pdf]

# Comparative genomics and pathogenicity analysis of two bacterial symbionts of entomopathogenic nematodes: The role of GroEL protein in virulence

Abraham Rivera-Ramírez [1] , Rosalba Salgado-Morales [2] , Alfredo Jimenez-Pérez [3] , Rebeca Martínez-Perez [2] , Blanca Ines García-Gómez [4] , and Edgar Dantan-González [2,*]

1 Posgrado en Ciencias, Instituto de Investigación en Ciencias Básicas y Aplicadas, Universidad Autónoma del Estado de Morelos, Av. Universidad 1001, Chamilpa, 62209 Cuernavaca, Morelos, México.

2 Laboratorio de Estudios Ecogenómicos, Centro de Investigación en Biotecnología, Universidad Autónoma del Estado de Morelos, Av. Universidad 1001, Chamilpa, 62209 Cuernavaca, Morelos, México.

3 Centro de Desarrollo de Productos Bióticos, Instituto Politécnico Nacional, Calle Ceprobi No. 8, San Isidro, Yautepec, 62739 Morelos, México.

4 Instituto de Biotecnología, Universidad Nacional Autónoma de México, Apdo. Postal 510-3, Cuernavaca, 62250, Morelos, México.

*Correspondence:edantan@uaem.mx ; Tel: +52-777-329-700

## Supplementary Figures

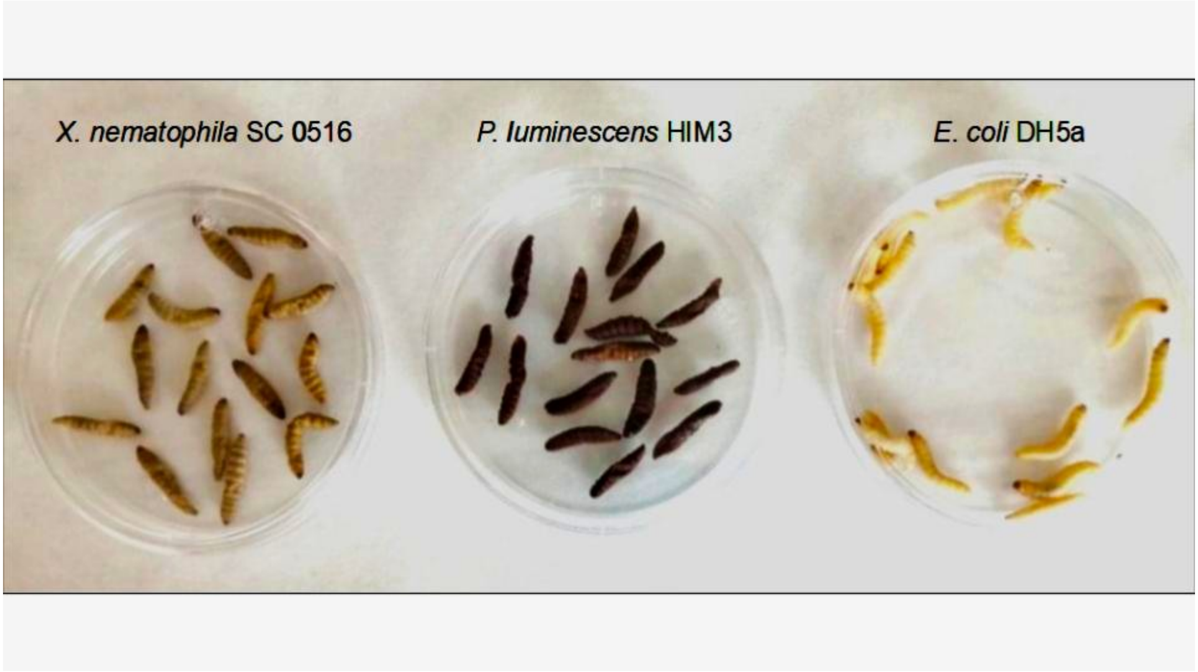


**Figure S1**. Phenotypes of *G. mellonella* larvae after injection *X. nematophila* SC 0516. From left to right: the phenotype of the fifth instar larva of *G. mellonella* infected with *X. nematophila* SC 0516, *P. luminescens* HIM3 and the *E. coli* DH5a (negative control) at 48 hours post infection.

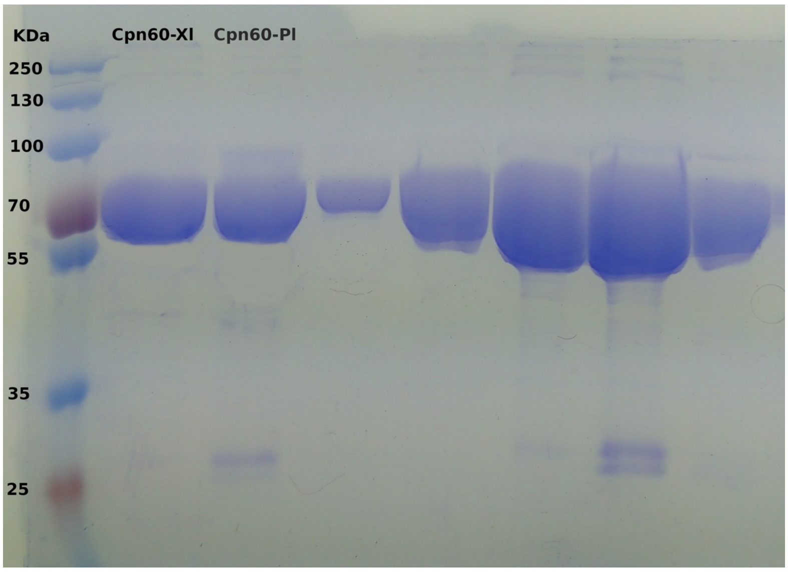


**Figure S2**. SDS-PAGE of purified GroEL proteins from *Xenorhabdus nematophila* SC 0516 (Cpn60-Xn) and *Photorhabdus luminescens* HIM3 (Cpn60-Pl). The gel was stained with Coomassie Blue and 5 µg of protein was loaded per well.

```
Cpn60-Xn        MAAKDVKFGNDARSKMLRGVNVLADAVKVTLGPKGRNVVLDKSFGAPVITKDGVSVAREI     60
Cpn60-Pl        MAAKDVKFGSDARVKMLRGVNILADAVKVTLGPKGRNVVLDKSFGAPAITKDGVSVAREI     60
                *********.*** *******:*************************.************

Cpn60-Xn        ELEDKFENMGAQMVKEVASKANDAAGDGTTTATVLAQAIVIEGLKAVAAGMNPMDLKRGI     120
Cpn60-Pl        ELEDKFENMGAQMVKEVASKANDAAGDGTTTATVLAQSIVTEGLKAVAAGMNPMDLKRGI     120
                *************************************:** *******************

Cpn60-Xn        DKAVVSAVEELKKLSVPCSDSTAIAQVGTISANSDETVGKLIAEAMDKVGKEGVITVEEG     180
Cpn60-Pl        DKAVVAAVAELKKLSVPCSDSTSIAQVGTISANSDETVGKLIAEAMDKVGKEGVITVEEG     180
                *****:** *************:*************************************

Cpn60-Xn        TGLEDELAVVEGMQFDRGYLSPYFINKPESGSVELENPYILLVDKKISNIRELLPVLEGV     240
Cpn60-Pl        TGLEDELDVVEGMQFDRGYLSPYFINKPENGSVELENPYILLVDKKISNIRELLPVLEGV     240
                ******* *********************.******************************

Cpn60-Xn        AKASKPLVIIAEDVEGEALATLVVNNMRGIVKVASVKAPGFGDRRKAMLQDIATLTNGTV     300
Cpn60-Pl        AKASKPLLIVAEDVEGEALATLVVNTMRGIVKVAAVKAPGFGDRRKAMLQDIAVLTNGNV     300
                *******:*:***************.********:******************.****.*

Cpn60-Xn        ISEEIGLELEKATLEDLGQAKRVVINKDTTTIIDGVGEEGAIAARVTQIRQQIEESTSDY     360
Cpn60-Pl        ISEEIGLELEKATLEDLGQAKRIVINKDTTTIIDGVGEEDAIAGRVAQINQQIKESTSDY     360
                **********************:****************.***.**:**.***:******

Cpn60-Xn        DREKLQERVAKLAGGVAVIKVGAATEVEMKEKRARVDDALHATRAAVEEGVVAGGGVALV     420
Cpn60-Pl        DREKLQERVAKLAGGVAVIKVGAATEVEMKEKRARVDDALHATRAAVEEGVVAGGGVALV     420
                ************************************************************

Cpn60-Xn        RVASAISGLTGENEDQNVGIRVAMRAMEAPMRQIVDNSGEEPSVVVNNVKAGENNYGYNA     480
Cpn60-Pl        RVAAAIAGLKGDNEDQNVGIRVAMRAMEAPLRQIVDNSGEEPSVIANSVKAGEGNYGYNA     480
                ***:**:**.*:******************:*************:.*.*****.******

Cpn60-Xn        TTEQYGDMIEMGILDPTKVTRSALQFAASIAGLMITTEAMVTDLPKDDKADLGAAGGMGG     540
Cpn60-Pl        TTEQYGDMIAMGILDPTKVTRSALQFAASIAGLMITTECMITDLPKDDKADLGAAGGMGG     540
                ********* ****************************.*:*******************

Cpn60-Xn        MGGMGGMM  548
Cpn60-Pl        MGGMGGMM  548
                ********
```

**(A)**

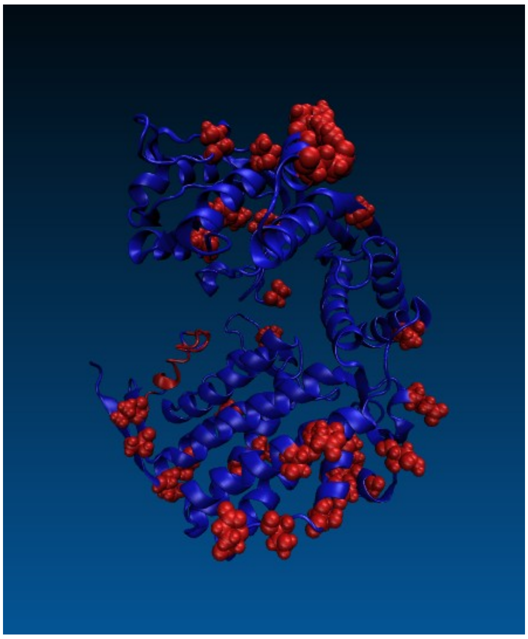

**(B)**

**Figure S3.** (A) Global alignment of the Cpn60-Xl and Cpn60-Pl sequences. The asterisks (*) below the sequences represent identical amino acids, (:) shows amino acids with similar physicochemical properties and (.) amino acids with different properties. (B) Mapping on the 3D structure of the GroEL protein of the 35 different substitutions (red) between Cpn60-Xl and Cpn60-Pl.
